# Injury Prevention in Amateur Soccer: A Nation-Wide Study on Implementation and Associations with Injury Incidence

**DOI:** 10.3390/ijerph16091593

**Published:** 2019-05-07

**Authors:** Angela Gebert, Markus Gerber, Uwe Pühse, Hanspeter Stamm, Markus Lamprecht

**Affiliations:** 1 Lamprecht und Stamm Sozialforschung und Beratung, Forchstrasse 212, CH-8032 Zurich, Switzerland; hp.stamm@lssfb.ch (H.S.); markus.lamprecht@lssfb.ch (M.L.); 2 Department of Sport, Exercise and Health, Sport Science Section, University of Basel, 4052 Basel, Switzerland; markus.gerber@unibas.ch (M.G.); uwe.puehse@unibas.ch (U.P.)

**Keywords:** injury prevention, preventive measures, prevention programmes, amateur soccer

## Abstract

Prevention programmes can reduce injury risk in amateur soccer. Hence, we examined the implementation of injury prevention in the real-world context of Swiss amateur soccer. In 2004 (*n* = 1029), 2008 (*n* = 705) and 2015 (*n* = 1008), a representative sample of Swiss amateur soccer coaches was interviewed by telephone about the frequency of injuries in their teams, the implementation of preventive measures and the use of injury prevention programmes. In the 2015 survey, 86.1% of amateur coaches stated that injury prevention is important and 85.3% of amateur coaches reported that they would implement some kind of preventive measures. The proportion of teams which performed a prevention programme according to minimal standards remained unchanged between 2008 (21.7%) and 2015 (21.9%), although a second prevention programme was made available in 2011. Only 8.6% of the 30+/40+ league teams, which are composed as a function of age, implemented a programme. Overall, the level of implementation of prevention programmes in this real-world context is still unsatisfactory. Offering an additional programme did not lead to a higher willingness to implement such programmes among the coaches. Concerted efforts are needed to remove barriers that hinder the use of such programmes, particularly among coaches of 30+/40+ league teams.

## 1. Introduction

Soccer is the most popular team sport in Switzerland [1]. Out of 6.2 million Swiss citizens aged between 15 and 74 years, about 480,000 were involved in playing soccer in 2013 [1]. While soccer is considered a meaningful leisure time activity that can enhance health [2,3], as a contact sport, it is also associated with an increased injury risk and therefore has a high socioeconomic impact [4,5]. The incidence of injury in amateur soccer ranges from 2.7 to 4.5 per 1000 h of training and from 12.3 to 24.7 per 1000 h of game play [6,7,8]. In order to reduce the injury risk, the Swiss Football Association (SFV) has promoted injury prevention strategies since 2004 [9]. More specifically, as part of their basic education and refresher courses, all Swiss soccer coaches are instructed to implement prevention programmes in their training plans. Furthermore, fair play measures were launched in 2007 by the SFV [10]. For instance, in low-level and junior leagues, a ranking-relevant penalty point system was introduced for red and yellow cards.

There is empirical evidence that prevention programmes can reduce injury risk in both amateur [11,12,13,14] and youth soccer [15,16,17]. However, not all studies have been able to detect a significant reduction in injury risk in the intervention group [18,19]. Hammes et al. [19] have attributed the lack of significant results to the low number of training sessions of some amateur soccer teams, which does not allow for neuromuscular adaptations. Alternatively, Steffen et al. [18] have assumed that the low compliance of teams and players may reduce the positive impact of prevention programmes. Generally, a high compliance of players with preventive measures is identified as being a key factor in the successful implementation of preventive strategies in soccer, and sports in general [20,21]. Furthermore, previous studies have highlighted the key role played by coaches in the promotion of preventive strategies [17,22,23].

A substantial limitation of existing research consists of the fact that most evidence is based on randomized controlled trials (RCTs) in which the programme implementation is prescribed and monitored by the researchers [14,24]. There is no doubt that RCTs should be seen as the “gold standard” in order to document programme efficacy, but studies with alternative study designs might provide valuable information used to obtain insight into the generalizability of the findings in real-world contexts, where preventive measures are carried out voluntarily and under less controlled circumstances [25]. For instance, Junge et al. [9] showed that the prevention programme “The 11” has been successfully implemented in Swiss amateur soccer, and that this programme is associated with a reduced injury incidence rate.

Given this background, the aim of the present study was to extend this study by Junge et al. [9] by exploring the current state and the development of injury prevention in Swiss amateur soccer by comparing retrospective survey data from 2004, 2008 and 2015. Moreover, we aimed to examine the association between the injury incidence of a team and the implementation of a prevention programme. In this manuscript the following research questions will be addressed: (1) How well are preventive measures implemented in Swiss amateur soccer, and are there any differences between leagues? (2) To what extent are specific prevention programmes implemented in Swiss amateur soccer, and are there any differences between leagues? (3) Is the implementation of prevention programmes by Swiss soccer coaches associated with a lower injury incidence rate among players?

## 2. Methods

### 2.1. Prevention Programmes

Since 2004, three prevention programmes have been launched in Swiss amateur soccer. “The 11” prevention programme was developed by FIFA as warm-up programme to reduce the most common soccer injuries and was integrated into coach education from 2004 onwards [9,26]. “The 11” includes ten exercises focusing on core and hamstring strength, balance and dynamic stabilisation as well as a fair play rule. “11+” is a revised version of “The 11” which was launched in 2009 and comprises 15 exercises, which are grouped into three parts [27]. The first part focuses on low speed running exercises and active stretching, while the second includes core and leg strength exercises and the third consists of moderate and high-speed exercises combined with planting and cutting movements. Additionally, all exercises of the second part are provided in three levels with increasing difficulty. There is evidence that “11+” is efficacious [11,12,14]. Since it is not obvious whether coaches will make a distinction between “The 11” and “11+”, these programmes have been recorded together as 11/11+. In addition, Suva “Sport Basics” (SSB) is a prevention programme developed for all ball sports, promoted by Suva (the Swiss National Insurance Fund) and launched in 2011 [28]. This programme has been integrated into coach education instead of “The 11”. It consists of six basic exercises which focus on strengthening the core and stabilisation of the axis of the leg. Additionally, four exercises with a higher difficulty level are provided for advanced athletes. “Sport Basics” has not been evaluated in a randomised controlled trial or interventional study.

### 2.2. Study Design

In May 2015, a retrospective survey was carried out with a representative sample of 1008 Swiss amateur soccer coaches regarding their use of injury prevention strategies and the frequency of injuries in their teams (response rate: 80.0%). Only coaches of amateur teams which consisted of players older than 14 years were included. In order to explore the development of coaches’ self-reported use of prevention strategies, data of two further surveys of Swiss amateur soccer coaches carried out in May 2008 (*n* = 705) and May 2004 (*n* = 1029) by Junge et al. [9] were included. Methods of the 2004 and 2008 surveys have been described in detail by Junge et al. [9]. The 2015 survey used the same methods as in 2008. However, questions on SSB were added. The telephone interviews were computer-assisted and fully structured. On average, an interview took 12 minutes in 2004, 20 minutes in 2008 and 21 minutes in 2015. As stated by the Art. 2 Human Research Act (HRA) and the Art. 25 Human Research Ordinance (HRO), ethical committee approval is not required for anonymised surveys. All procedures performed in this study were in accordance with the ethical principles stated in the Declaration of Helsinki.

### 2.3. Questionnaire

The questionnaire was developed by Junge et al. [9] in light of the well-established consensus statement of Fuller et al. [29]. First, the coaches had to answer some basic questions about their teams (league, team size) and their training (frequency, level of attendance).

Second, the questionnaire included some questions about injury prevention. With respect to preventive measures, coaches who stated that they would implement such measures were asked to name all (unprompted questioning). These answers were categorised by the interviewers. Furthermore, the coaches were asked whether they knew and used the prevention programmes 11/11+ and SSB and how frequently they taught these programmes or some exercises from them. For the more in-depth analyses, teams were divided into four groups: those that implemented SSB according to minimal standards, those that implemented 11/11+ according to minimal standards, those that implemented both programmes according to minimal standards and those that implemented parts of a programme (but not according to minimal standards) or had never performed a programme. For the purpose of the present study, implementation of a programme according to minimal standards was accomplished if the coach used at least three exercises of a programme per session at least once per week over at least six months. With respect to specific types of exercises, all coaches had to mention how frequently they performed one-legged coordination and balance exercises, core strength exercises, hamstring strength exercises and jumping power exercises with their teams. In the analyses, a distinction was made between coaches who regularly (frequently, each training) and not regularly (never, rarely, sometimes) implemented a specific type of exercise.

Third, the procedure for the recording of injuries was strictly predetermined in order to improve the accuracy of injury reports. The coaches were asked about the number of games played in the previous four weeks and they were then asked to remember the last game by mentioning the opponent and reporting all related injuries sustained by their players. For each injury mentioned detailed information about body region, type, contact, foul play, severity and medical attention was recorded. The same procedure was repeated back in time for each game played during the four weeks before the interview. Moreover, the coaches had to report the number of training injuries which occurred during the four weeks before the interview and had to provide detailed information about each. 

### 2.4. Statistical Analysis

Descriptive data were presented as the mean and standard deviations (SD), and as percentages with 95% confidence intervals (95% CI). Statistical methods applied were χ^2^ statistics and the significance level was set to 5%. Injury incidences were calculated as injuries per 1000 h of training and injuries per 1000 h of game play. For the calculation of game injury incidence five games were considered at most in order to reduce recall bias. Following Knowles et al. [30], 95% CI were provided for injury incidences and were calculated as:

(1) $$\mathrm{Incidence}\;\mathrm{rate}\pm 1.96\times \sqrt{\left(\mathrm{number}\;\mathrm{of}\;\mathrm{injuries}\right)}÷\left(\mathrm{person}\text{-}\mathrm{time}\;\mathrm{at}\;\mathrm{risk}\right)$$

Moderate overlap between the bars of the 95% CI (no more than half of each bar) was the criterion for statistically significant differences of injury incidences at a *p*-value of 0.05 [31]. The statistical analysis was performed using SPSS 24.0 for Mac (SPSS, Chicago, Illinois, USA) and Excel 2001 for Mac (Microsoft, Redmond, WA, USA).

## 3. Results

### 3.1. Injury Prevention in Swiss Amateur Soccer

In the 2015 survey, a majority of coaches confirmed that injury prevention plays an important role in their training plans (86.1%, 95% CI 84.0–88.3). However, the number of affirmative answers was slightly higher in the previous surveys (2008: 90.9%, 95% CI 88.7–93.0; 2004: 89.3%, 95% CI 87.4–91.2). In accordance with this, a high percentage of coaches reported that they would implement specific measures to prevent the injury of their players (85.3%, 95% CI 81.9–86.3). In 2008, this percentage was slightly higher (89.8%, 95% CI 87.6–92.0), while in 2004 it was similar (84.1%, 95% CI 81.9–86.3). There were significant differences by leagues (χ^2^[5,1006] = 64.261, *p* < 0.001), indicating that preventive measures were less frequently implemented in 30+/40+ league teams (64.7%, 95% CI 56.0–73.4) compared to other teams (male second and third amateur leagues 85.3%, 95% CI 79.2–91.4; male fourth and fifth amateur leagues 79.9%, 95% CI 74.7–85.2; male 16–20 years 93.0%, 95% CI 89.5–96.4; male 14–15 years 92.0%, 95% CI 88.5–95.5; female all levels 89.9%, 95% CI 84.0–95.8).

Furthermore, Table 1 shows which preventive measures the coaches implemented. In the 2015 survey, the most commonly reported measures were warm-ups, stretching, general strength training and core strength training, whereas specific prevention programmes were rarely mentioned. 11/11+ was less frequently mentioned in 2015 compared to 2008, but the percentage of coaches who implemented general strength training and core strength training had increased significantly. Warm-up and stretching were less frequently reported in the 2015 survey compared to the 2004 survey.

### 3.2. Implementation of Prevention Programmes

When the coaches were asked whether they knew SSB or 11/11+, 43.0% (95% CI 39.9–46.0) stated that they knew SSB and 48.4% (95% CI 45.3–51.5) stated that they knew 11/11+. All in all, 33.2% (95% CI 30.3–36.1) of coaches stated that they knew both programmes. The percentage of coaches who knew 11/11+ did not differ from 2008 (46.2%, 95% CI 42.5–29.9). 16.3% (95% CI 14.0–18.5) of the coaches reported that they would implement SSB or at least a selection of exercises with their team and 21.8% (95% CI 19.3–24.4) of the coaches reported that they would implement 11/11+ or at least a selection of exercises with their team. Moreover, 18.2% (95% CI 15.8–20.5) reported that they would implement both programmes or at least particular exercises of them.

Coaches who implemented a prevention programme reported that on average they instruct 3.9 (SD = 1.9) exercises per session for a mean duration of 13.7 minutes (SD = 7.8). Furthermore, 56.9% (95% CI 53.0–60.8) of them reported that they would implement this prevention programme at least once a week. Taken together, the programme implementation did not differ significantly from 2008 (3.7 exercises, 13.5 min, 60.4% once per week).

**Figure 1:**
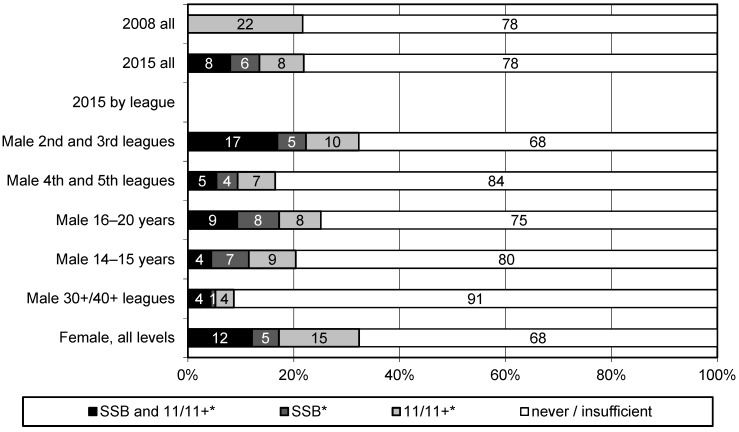
Implementation of prevention programmes according to minimal standards in Swiss amateur soccer by leagues (in percent). * Implementation according to minimal standards: at least three exercises of a programme per session at least once per week over at least six months. SSB: Suva “Sport Basics”, 11/11+: FIFA “The 11” and “11+”.

As shown in Figure 1, the percentage of teams in which a prevention programme was carried out according to minimal standards did not change between 2008 (21.7%, 95% CI 18.6–24.8) and 2015 (21.9%, 95% CI 19.3–24.5) despite the fact that in 2015 an additional programme (SSB) was available. Coaches of 30+/40+ teams less frequently implemented prevention programmes according to minimal standards (8.6%, 95% CI 3.5–13.7) compared to coaches of other teams (male second and third leagues 32.3%, 95% CI 24.3–40.3; male fourth and fifth leagues 16.5%, 95% CI 11.6–21.4; male 16–20 years 25.2%, 95% CI 19.4–31.0; male 14–15 years 20.4%, 95% CI 15.1–25.7; female all levels 32.3%, 95% CI 23.1–41.5). 

### 3.3. Association between Injury Incidence and the Implementation of a Prevention Programme

In 2015, the coaches reported 1076 injuries which happened during nearly 4000 amateur soccer games, and 525 injuries which occurred during about 180,000 h of training. The overall injury incidence was 16.5 (95% CI 15.5–17.4) injuries per 1000 h of competitive playing and 2.9 (95% CI 2.6–3.1) injuries per 1000 h of training. Injury causes and injury characteristics have been described by Gebert et al. [32].

**Figure 2:**
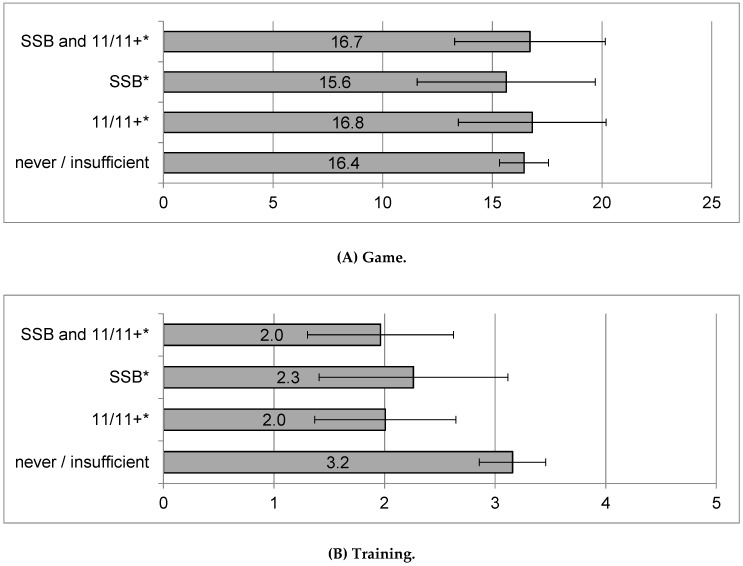
Injury incidence per 1000 h of game play (**a**) and training (**b**) by implementation of a prevention programme according to minimal standards in 2015 (including 95% CI). * Implementation according to minimal standards: at least three exercises of a programme per session at least once per week over at least six months. SSB: Suva “Sport Basics”, 11/11+: FIFA “The 11” and “11+”.

Implementing a prevention programme according to minimal standards was not associated with a lower injury incidence during games, but minimal implementation of 11/11+ or of both programmes was significantly associated with a 37.5% lower injury incidence during training (see Figure 2).

Regarding the association between injury incidence and specific exercises, one-legged coordination and balance training was significantly associated with a lower injury rate. Teams which regularly performed one-legged coordination and balance exercises had an 18.0% lower game injury incidence (15.0, 95% CI 13.8–16.3 versus 18.3, 95% CI 16.7–19.9) and a 28.6% lower training injury incidence (2.5, 95% CI 2.2–2.8 versus 3.5, 95% CI 3.0–3.9). However, with regard to exercises focusing on core strength, strengthening of the hamstrings and jumping power, no significant associations with injury incidence were found.

## 4. Discussion

The present study has analysed the implementation of injury prevention in amateur soccer based on three surveys (2004, 2008 and 2015) conducted with representative samples of Swiss amateur soccer coaches. Furthermore, the association between injury incidence and the implementation of prevention programmes has been examined.

The principal finding was that 22% of amateur soccer coaches implemented a prevention programme according to minimal standards if they have learned it in the course of their coaches’ education. Although various prevention programmes have been available for several years, the percentage of soccer coaches who have implemented a prevention programme did not differ between the 2008 and 2015 surveys. This result suggests that a wider range of programmes does not automatically lead to a higher willingness to implement them. Bogardus et al. [33] have identified motivation, time and skill requirements, compliance and costs as barriers to the implementation of anterior cruciate ligament injury prevention programmes. Consequently, further measures are needed to reduce such barriers and to convince coaches of the importance of consistent implementation of prevention programmes.

Nevertheless, 86% of coaches actually confirmed in the 2015 survey that including injury prevention in the training programme is important [9]. Several studies highlight coaches’ high levels of compliance with injury prevention [20,34]. It can be assumed that a coach influences the compliance of his or her players [20], which represents an important factor in the effectiveness of injury prevention programmes [17,21,34,35]. Interestingly, coaches of 30+/40+ teams were less willing to implement preventive measures and prevention programmes than coaches of other teams. This finding is alarming, as research shows that the risk of injury increases with age [32,36] and that injury incidence is extremely high among Swiss 30+/40+ league players [32]. Possible reasons for the lower willingness to implement preventive measures could be structural problems like the low frequency of training sessions [19] or the lack of a (well-educated) coach [9].

In the 2015 survey, the most frequently mentioned preventive measures were warm-ups, stretching, general strength training and core strength training. While warm-ups and stretching were less frequently reported compared to the 2004 survey, the coaches more frequently mentioned general strength training and core strength training. We assume that warm-ups are still implemented by (nearly) all coaches, but have become so established in everyday training that the coaches decreasingly associate this measure with prevention [37]. Even if there is no evidence for the preventive effect of stretching, this measure is often used by coaches and soccer players [38]. The fact that strength training (general and core) was more frequently mentioned as a preventive measure in the 2015 survey suggests a positive development. However, the results also indicate that coaches were less reminiscent of specific prevention programmes in 2015 than they were in 2008.

Based on the results of the present study no causal effects of prevention programmes on injury incidence can be deduced. We could show that teams which implemented 11/11+ according to minimal standards had a 38% lower training injury incidence than teams which did not implement a prevention programme. It is plausible that the implementation of a prevention programme during training has led to a reduction of injury incidence, since during the performance of these exercises no injuries did occur. Furthermore, injury incidence during games did not differ between teams performing a prevention programme and teams not performing a programme. One possible explanation for this finding is that coaches of injury-prone teams are more likely to seriously implement a prevention programme. Another conclusion could be that performing a programme once a week may not be enough to cause substantial preventive effects with respect to game injuries [14,19,39]. Furthermore, the results of the present study indicate that regularly performing one-legged coordination and balance exercises may be more effective in preventing both training and game injuries than other exercises. Further research is needed in this regard.

### Limitations

The design of the present study is associated with some methodological weaknesses which have also been discussed previously by Junge et al. [9]. A considerable limitation is that the injury data was collected by interviews with coaches, which might cause memory effects and reporting bias. Ekegren et al. [40] have concluded that sports coaches are likely to underestimate injury rates. However, injury incidences calculated in the present study accorded well with previous research [6,7,8]. Additionally, implementing a prevention programme according to minimal standards required that a team had performed the programme for at least six months, at least once per week, and with at least three exercises per session. This lower limit was chosen to account for the fact that many low-level amateur soccer teams only train once per week. Setting the limit to an implementation of two or three times per week could have strengthened the association between injury incidence and the implementation of prevention programmes [14,39], but would have disregarded real conditions and systematically eliminated many teams.

## 5. Conclusions

The results of the present study have practical significance with regard to the development of preventive strategies in Swiss amateur soccer. It could be shown that Swiss amateur soccer coaches have a positive attitude towards injury prevention. However, the implementation rate of prevention programmes needs to be further optimised. Therefore, a main objective must be to systematically remove barriers that impede the implementation of prevention programmes, especially in teams of 30+/40+ leagues.

## Figures and Tables

**Table 1:** Implementation of preventive measures: preventive measures reported by the coaches (unprompted questioning) by percentage of coaches who reported taking preventive measures (including 95% confidence interval (CI)).

Preventive Measures	2004 % (95% CI)	2008 % (95% CI)	2015 % (95% CI)
Suva Sport Basics	–	–	5.7 (4.2–7.3)
The 11/11+	–	25.6 (22.2–29.0)	6.5 (4.9–8.2)
Warm–up	80.7 (78.0–83.3)	72.4 (68.9–75.8)	67.4 (64.2–70.5)
Stretching	74.9 (72.0–77.8)	48.0 (44.1–51.9)	47.8 (44.4–51.1)
Cool down	35.1 (31.9–38.3)	12.0 (9.5–14.5)	12.9 (10.7–15.2)
Wearing shin guards	21.6 (18.9–24.4)	13.4 (10.8–16.1)	9.8 (7.8–11.8)
General strength training	16.2 (13.7–18.7)	13.3 (10.6–15.9)	24.2 (21.4–27.1)
Massage	11.5 (9.3–13.6)	6.2 (4.3–8.0)	4.4 (3.1–5.8)
Information	11.0 (8.9–13.1)	3.3 (1.9–4.7)	3.4 (2.2–4.6)
Core strength training	10.0 (8.0–11.9)	7.4 (5.4–9.5)	20.9 (18.1–23.6)
Cardiorespiratory fitness training	10.0 (8.0–11.9)	4.4 (2.8–6.0)	10.1 (8.1–12.2)
Rehabilitation and complete recovery	5.3 (3.8–6.8)	1.7 (0.7–2.8)	3.3 (2.1–4.5)
Fair play	4.5 (3.1–5.9)	2.1 (0.9–3.2)	3.6 (2.4–4.9)
Adjusting footwear	4.2 (2.8–5.5)	1.3 (0.4–2.1)	1.9 (1.0–2.8)
Other measures	14.1 (11.8–16.4)	11.2 (8.8–13.7)	18.1 (15.5–20.6)
Number of coaches	864	633	858
